# Supplementary material for: Evidence for persistence of the SHIV reservoir early after MHC haploidentical hematopoietic stem cell transplantation
Source: Nat Commun. 2018 Oct 25;9:4438. doi: 10.1038/s41467-018-06736-7 (PMC6202377; doi:10.1038/s41467-018-06736-7)
Supplement: Supplementary file 1 — Supplementary Information [file 41467_2018_6736_MOESM1_ESM.pdf]

Evidence for Persistence of the SHIV Reservoir Early After MHC Haplo-identical Hematopoietic Stem Cell Transplantation

Colonna et al.

Supplementary Information

Animal ID	Donor/Recipient origin	Tissue	Sample Type	Purity by Flow
R.702	A01+	Spleen	CD4+ T cells	100%
R.702	A01+	Rectum	CD4+ T cells	100%
R.703	A01-	Spleen	CD4+ T cells	97.1
R.703	A01-	Spleen	CD4+ Non-T cells	92.3
R.703	A01+	Spleen	CD4+ T cells	97.1
R.703	A01+	Spleen	CD4+ Non-T cells	99.8
R.703	A01-	Bone Marrow	CD4+ T cells	92.3
R.703	A01+	PBMC	CD4+ Non-T cells	100%
R.703	A01-	Lung	CD4+ T cells	88.4%
R.703	A01+	Lung	CD4+ T cells	60.4%
R.703	A01+	Lung	CD4+ Non-T cells	100%

Supplementary Table 1. Post-sorting quality control of donor versus recipient CD4+ cells. Following cell sorting, post-sort flow cytometry-based quality control analysis was performed for all samples where enough cells were available to assess the purity of donor-derived (A*01+), and recipient-derived (A*01-) CD4+ T (CD3+ CD4+) and CD4+ non-T (CD3- CD4+) cells.

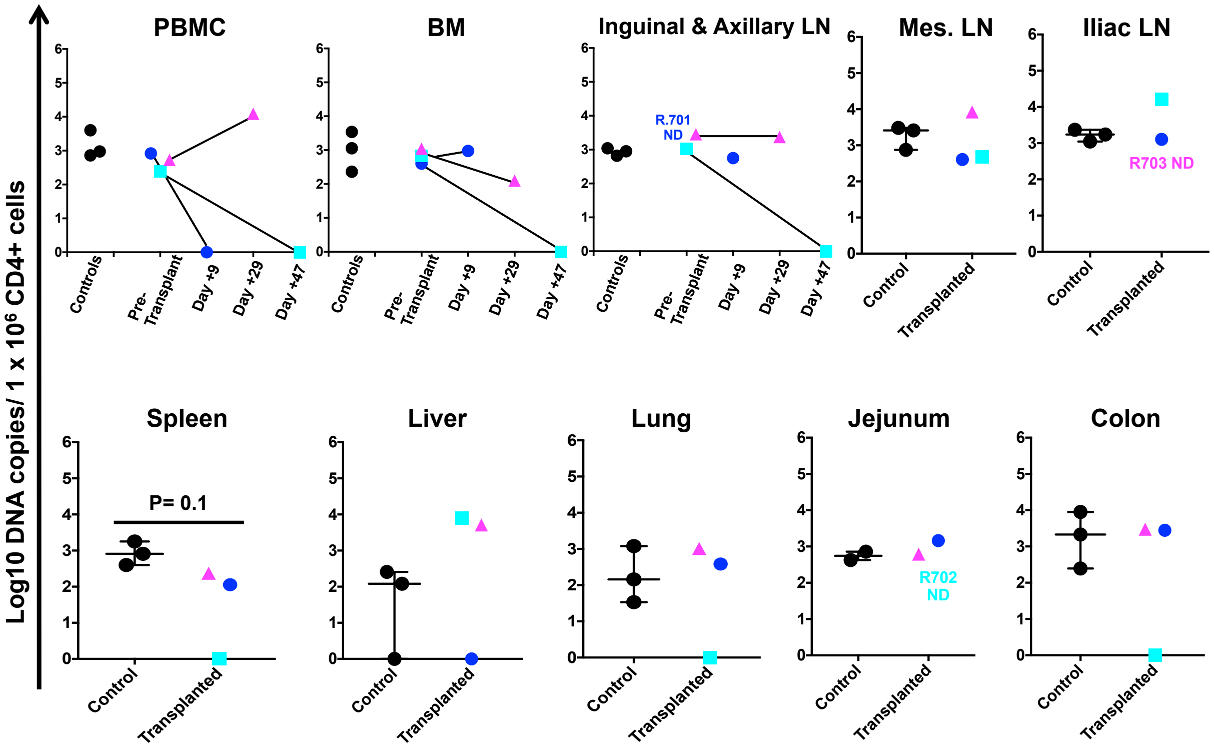

Supplementary Figure 1. SHIV-C DNA in transplant recipients and controls normalized to proportion of CD4+ T cells. The total SHIV DNA copy number was normalized to $1x10^6$ CD4+ T cells in 10 tissues in which the percentage of CD4+ T cells could be measured by flow cytometry (PBMC, BM, pooled inguinal and axillary LN, mesenteric LN, iliac LN, spleen, liver, lung, jejunum, colon). Error bars represent the mean with SEM.

a)

Animal ID	R.707
Stem Cells Source	Total BM
Donor Relationship and Matching	Half-sibs, Haplo-identical
Conditioning Regimen	300 cGy TBI + Cyclophosphamide (14.5 mg/kg on Day -5 and -4; 30 mg/kg on Day +3 and +4)
TNC/Kg	0.96×10^8
CD34/ Kg	2.89×10^6
CD3/ Kg	28.5×10^6

b)

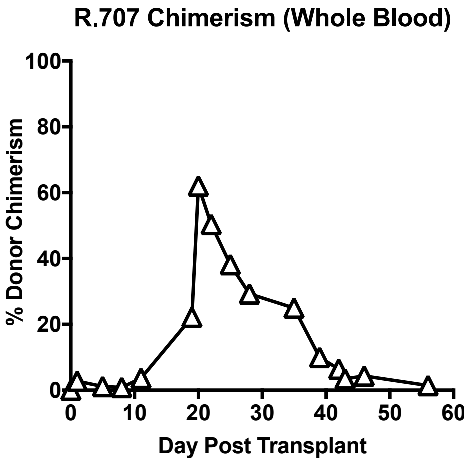

Supplementary Figure 2. Transplant characteristics and percent donor chimerism of animal ID R.707. a) Conditioning regimen and transplant characteristics of Animal ID R.707. **b**) Whole blood molecular chimerism analysis following reduced intensity conditioning and treatment with Post-Transplant Cyclophosphamide (PT-Cy).
